# Protocol for potent activation of T cells using BPI-stimulated murine bone marrow-derived cells

**DOI:** 10.1016/j.xpro.2026.104519

**Published:** 2026-04-24

**Authors:** Christina Pfab, Jonas M. Holzinger, Sarah Hirsch, Katharina U. Roithmeier, André Gessner, Sigrid Bülow

**Affiliations:** 1 Institute of Medical Microbiology and Hygiene Regensburg, University of Regensburg, Regensburg 93053, Germany; 2 Institute of Clinical Microbiology and Hygiene, University Hospital Regensburg, Regensburg 93053, Germany

**Keywords:** Cell culture, Cell isolation, Cell-based Assays, Immunology, Model Organisms

## Abstract

*In vitro* T cell activation and differentiation are fundamental techniques in immunological research. Here, we present a protocol for the potent activation of T cells using bactericidal/permeability-increasing protein (BPI)-stimulated murine bone marrow-derived cells (BMDCs). We describe steps for preparing supernatants harvested from BPI-stimulated BMDCs (SN BPI). We then detail procedures for purification of (naive) CD4^+^ T cells from murine spleens and peripheral lymph nodes and their consecutive polyclonal activation in the presence of SN BPI.

For complete details on the use and execution of this protocol, please refer to Bülow et al.^1^

## Highlights

• Guidance for cultivating murine bone marrow-derived cells (BMDCs)
• Instructions to stimulate BMDCs using bactericidal/permeability-increasing protein
• Steps for the isolation of murine T cells
• Procedures for T-cell activation in the presence of stimulatory BMDC supernatants

## Abstract (editor highlights)

Publisher’s note: Undertaking any experimental protocol requires adherence to local institutional guidelines for laboratory safety and ethics.

## Graphical abstract

**Figure:**
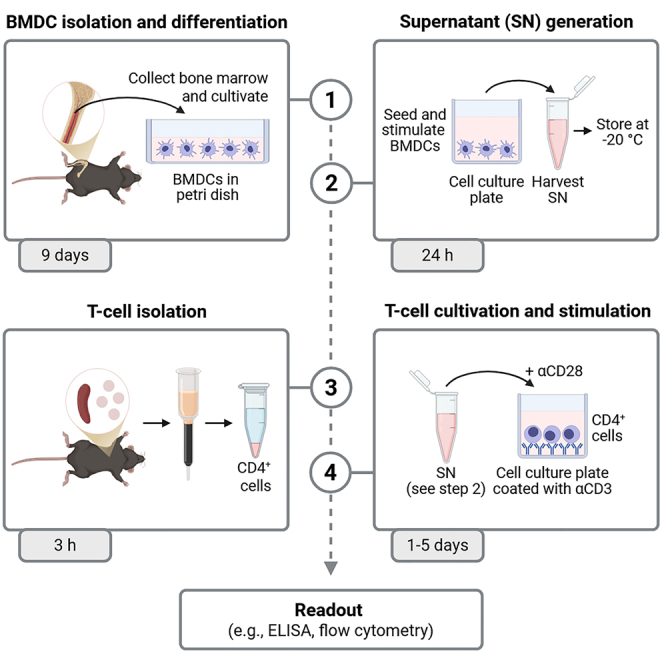

## Before you begin

Antigen-presenting cells (APCs), such as dendritic cells (DCs), monocytes and macrophages (MΦ), are capable of activating and differentiating T-cells. This process requires three essential signals: (1) the interaction between the major histocompatibility complex (MHC) and the T-cell receptor (TCR), (2) the engagement of the B7 complex (CD80 and CD86) on the APCs with CD28 on the T-cells; and (3) soluble factors in the form of fingerprint cytokines, which are required for the differentiation of specific T-cell subsets. This protocol was created to investigate the influence of these soluble factors derived from APCs on T-cell populations. For this purpose, APCs are stimulated with pathogen- or danger-associated molecular patterns (PAMPs or DAMPs, respectively) such as bactericidal/permeability-increasing protein (BPI) to secrete cytokines. Following stimulation, the supernatant (SN) containing these cytokines is collected in order to activate and differentiate T cells. This protocol places special emphasis on the soluble factors present in the SN of stimulated APCs. Thus, the direct interaction between BMDCs and T cells (signals 1 and 2 of T-cell activation) is replaced by adding αCD3 and αCD28 antibodies to the cell culture.

In this protocol, APCs are represented by GM-CSF-cultured bone marrow-derived cells (BMDCs), which contain both MΦ and DC populations.^2^ The induction of cytokine secretion in these cells is illustrated using BPI as an example. BPI was recently discovered as a neutrophil-derived DAMP towards BMDCs.^1^ Thereby, BPI is capable of inducing the differentiation of T_H22_ via a DC T-cell axis.^1^ The SN generated by BPI-stimulated BMDCs activates CD4^+^ T cells in the presence of signal 1 and 2. Since this SN contains interleukin (IL)-6 and tumor necrosis factor (TNF), the exposed CD4^+^ T cells differentiate preferentially into T_H22_ cells. Depending on the scientific question, this protocol can be modified, e.g., by choosing different APCs or T-cell subsets as well as various PAMPs and/or DAMPs. In this case, the reagents must be adapted accordingly.

Before starting the experiments, ensure the reagents indicated in the key resources table and the materials are available.

### Innovation

The isolation and *in vitro* stimulation of CD4^+^ T cells is an important method in T-cell immunology. This protocol outlines a comprehensive, and reproducible workflow combining several established methods to specifically evaluate the influence of soluble factors derived from BMDCs on CD4^+^ T cells. It incorporates several essential processes, including the generation and stimulation of BMDCs adapted from Lutz et al.^3^, the preparation of conditioned SN, the isolation of (naïve) CD4^+^ T cells via magnetic-activated cell sorting (MACS), and subsequent culture of the T cells under defined stimulatory conditions. Although each step is based on well-established *in vitro* techniques, integrating these steps into a single protocol makes the approach more practical and accessible. Although various PAMPs and DAMPs can be used accordingly as stimulants for APCs, potent T-cell activation is achieved by applying BPI.

### Institutional permissions

Mice were euthanized to harvest organs and tissues for scientific purposes in accordance with the German Animal Welfare Act (TierSchG). Therefore, no further approvals were required in our study. However, researchers should obtain necessary approvals if required by the respective national, local and/or institutional guidelines.

## Key resources table

**Table:** 

REAGENT or RESOURCE	SOURCE	IDENTIFIER
**Antibodies**		

BD Pharmingen™ Purified Rat Anti-Mouse CD16/CD32 (Mouse BD Fc Block™), clone 2.4G2 (RUO); 1:250 dilution	BD Biosciences	Cat# 553142 RRID:AB_394657
CD11c Antibody, anti-mouse, REAfinity™, VioBlue, clone REA754; 1:50 dilution	Miltenyi Biotec	Cat# 130-110-706 RRID:AB_2654712
CD11b Antibody, anti-mouse, REAfinity™, VioGreen, clone REA592; 1:50 dilution	Miltenyi Biotec	Cat# 130-113-811 RRID:2726328
BD Pharmingen™ Purified NA/LE Hamster Anti-Mouse CD3e, clone 145-2C11 (RUO); 1:50 dilution	BD Biosciences	Cat# 553057 RRID:AB_394590
BD Pharmingen™ Purified NA/LE Hamster Anti-Mouse CD28, clone 37.51 (RUO); 1:125 dilution	BD Biosciences	Cat# 553294 RRID:AB_394763
CD3 Antibody, anti-mouse, REAfinity™,FITC, clone REA606; 1:400 dilution	Miltenyi Biotec	Cat# 130-119-758 RRID:AB_2751822
BD Pharmingen™ PerCP Rat Anti-Mouse CD4; clone RM4-5 (RUO); 1:100 dilution	BD Biosciences	Cat# 553052 RRID:AB_394587
CD25 Antibody, anti-mouse, REAfinity™, APC, clone REA568; 1:50 dilution	Miltenyi Biotec	Cat# 130-120-767 RRID:AB_2752188
BD Pharmingen™ FITC Rat Anti-Mouse CD44, clone IM7 (RUO); 1:200 dilution	BD Biosciences	Cat# 553133 RRID:AB_2076224
CD44 Antibody, anti-mouse, PE-Vio 770 clone IM7.8.1; 1:10 dilution	Miltenyi Biotec	Cat# 130-102-377 RRID:AB_2658187
CD62L monoclonal antibody, PE, clone MEL-14; 1:200 dilution	Thermo Fisher Scientific	Thermo Fisher Scientific Cat# MA5-17802, RRID:AB_2539186
CD62L Antibody, anti-mouse, VioBlue, clone: MEL14-H2.100; 1:10 dilution	Miltenyi Biotec	Cat# 130-102-425 RRID:AB_2660519

**Chemicals, peptides, and recombinant proteins**		

Ammonium chloride (NH_4_Cl)	Merck	Cat# 1011451000
Potassium hydrogen carbonate (KHCO_3_)	Carl Roth	Cat# 9437.2
EDTA	Invitrogen	Cat# 15575-020
G418 sulfate	Thermo Fisher Scientific	Cat# 10131035
HEPES	Sigma	Cat# H0887
Sodium azide (NaN_3_)	Sigma	Cat# CDS003079
β-Mercaptoethanol	AppliChem	Cat# A1108

**Experimental models: Organisms/strains**		

C57BL/6J, 12**–**16 weeks old, male	The Jackson Laboratory	RRID:IMSR_JAX:000664

**Critical commercial assays**		

Naive CD4^+^ T-Cell Isolation Kit, mouse	Miltenyi Biotec	Cat# 130-104-453
CD4^+^ T-Cell isolation Kit, mouse	Miltenyi Biotec	Cat# 130-104-454
IL-22 ELISA Kit	Thermo Fisher	Cat# 88-7422-88

**Other**		

Dulbecco’s phosphate-buffered saline	Sigma Aldrich	Cat# D8537
Heat-inactivated fetal calf serum (FCS)	Sigma Aldrich	Cat# F7524
Penicillin-Streptomycin	PAN-Biotech	Cat# P06-07100
StableCell™ RPMI 1640	Thermo Fisher	Cat# R2405
BPI	Bülow et al.^1^	N/A
GM-CSF supernatant	Lutz et al.^4^ and Robinson et al.^5^	N/A
Viability Staining Solution	Thermo Fisher	Cat# 00-6993-50
Cell strainer (70 μm)	Sarstedt	Cat# 83.3945.070
MidiMACS separator	Miltenyi Biotec	Cat# 130-042-302
MACS MultiStand	Miltenyi Biotec	Cat# 130-042-303
LS Columns	Miltenyi Biotec	Cat# 130-042-401
Pre-Separation Filters (30 μm)	Miltenyi Biotec	Cat# 130-041-407
FACS Aria II	BD Biosciences	N/A
BD FACSCanto II	BD Biosciences	N/A
Luminex 100 system	Luminex corp.	N/A
Falcon 96-well Polystyrene Microplates	Corning	Cat# 353072
Petri dish 100 × 15 mm	Sarstedt	Cat# 82.1473
Sterile gauze swabs, e.g., ES gauze swabs	Hartmann	Cat# 401723
Ethanol-based skin disinfectant, e.g., Softasept N	Braun	Cat# 3887138
Cell culture flask, T-25, surface: Standard, filter cap	Sarstedt	Cat# 83.3910.002
Cell culture flask, T-175, surface: Standard, filter cap	Sarstedt	Cat# 83.3912.002
Discardit II (5 mL syringe)	BD	Cat# 309050
100 Sterican (20 G needle)	Braun	Cat# 4657519
100 Sterican (27 G needle)	Braun	Cat# 4657705 B
Millipore Steritop Vacuum Bottle-Top Filter (0.2 μm)	Millipore	Cat# S2GVT05RE
Laminar air flow cabinet	BDK Luft- und Reinraumtechnik	N/A
CO_2_ incubator	Thermo Scientific	N/A
Microscope Leica DM IL	Leica Microsystems	N/A
Pipette controller	Integra	N/A
Serological pipette, 10 mL	Sarstedt	N/A

**Software and algorithms**		

GraphPad Prism, version 7	GraphPad Software	N/A
FlowJo v.10	BD Biosciences	N/A
LiquiChip Analyzer Software	QIAGEN	N/A

## Materials and equipment

**Table:** FACS buffer

REAGENT	Final concentration	Amount
NaN_3_ (10% w/v in H_2_O)	0.1%	5 mL
FCS (heat inactivated)	1%	5 mL
PBS	N/A	500 mL
TOTAL	N/A	510 mL

Store at 4°C for up to 3 months.

**Table:** MACS buffer

REAGENT	Final concentration	Amount
EDTA	2 mM	2 mL
FCS (heat inactivated)	0.5%	2.5 mL
PBS	N/A	500 mL
TOTAL	N/A	504.5 mL

Store at 4°C for up to 3 months.

**Table:** Medium (for BMDCs and T-cells)

REAGENT	Final concentration	Amount
Penicillin/Streptomycin	1% (100 U/mL/100 μg/mL)	5 mL
L-Glutamine	1% (2 mM)	5 mL
β-Mercaptoethanol	0.1% (50 μM)	0.5 mL
FCS (heat inactivated)	10%	50 mL
RPMI 1640	N/A	500 mL
TOTAL	N/A	565 mL

Store at 4°C for 2**–**4 weeks.

**Table:** Red blood cell lysis buffer, pH 7.2–7.3 (sterile filtered, 0.2 μm)

REAGENT	Final concentration	Amount
NH_4_Cl	150 mM	8.02 g
KHCO_3_	10 mM	1.00 g
EDTA	0.1 mM	1 mL
Ultrapure water	N/A	1 L
TOTAL	N/A	1 L

Store at 4°C for up to 3 months.

## Step-by-step method details

**CRITICAL:** Sterile conditions must be adhered to.

### BMDC isolation and differentiation

**Timing: 7 days**

The aim of this protocol is to stimulate T cells with soluble factors derived from BMDCs. Therefore, BMDCs are generated from murine bone marrow and, once matured, stimulated to consecutively harvest the SN. The following section is adapted from Lutz et al.^3^

***Note:*** For additional guidance, please refer to Problem 1 in the troubleshooting section.

**CRITICAL:** Adherence to sterile conditions is paramount.

1. Prepare primary BMDC culture.
  ***Note:*** Culture of bone marrow cells using GM-CSF is a suggested method to generate BMDCs. However, IL-4 or FLT-3 may be added in dependence of the scientific interest.^4^.
  Before starting bone marrow cell isolation, warm sterile PBS and medium to 37°C.
  a. Sacrifice 12**–**16 weeks old male C57BL/6J mice by carbon dioxide asphyxiation or cervical dislocation.
    ***Note:*** Use sterilized instruments for the following procedures. In order to inactivate bacterial endotoxin, dry heat sterilization at 180°C is preferred.
    ***Optional:*** Mice of other age and both genders can be used but primary BMDCs derived from these animals need to be tested for proper responsiveness to the treatment. Consistently using mice of the same age will improve experimental reproducibility.
  b. Sterilize the hind legs using an ethanol-based skin disinfectant.
  c. Harvest femur and tibia from one or both hind legs.
    ***Note:*** Harvesting femur and tibia from one leg yields approximately 4 × 10^7^ cells but may vary between individual mice.
  d. Manually remove muscle and connective tissue from the bones using sterile gaze pads and store femur and tibia in empty tubes.
    ***Note:*** Work in a laminar air flow cabinet for all further steps.
  e. Prepare syringe and needle:
    i. Draw 5 mL of sterile PBS into a 5 mL syringe using a 20 G needle.
    ii. Discard the 20 G needle and replace it with a 27 G needle.
  f. Rinse the bone marrow cells.
    i. Separate femur and tibia.
    ii. Open each bone at both the proximal and distal ends.
    iii. Insert the syringe containing PBS with the 27 G needle into the bone.
    iv. Flush the bone marrow into a 50 mL tube.
  g. Centrifuge (8 min, 300 × *g*, room temperature).
  h. Discard the supernatant.
  i. Resuspend the cells in 1 mL medium, then add 9 mL of medium.
  j. Count cells at a 1:10 dilution.
  k. Seed bone marrow cells.
    i. Seed 4 × 10^6^ bone marrow cells per petri dish.
    ii. Add medium containing GM-CSF at a concentration of 200**–**400 U/mL^4^ to a total volume of 10 mL.
      ***Note:*** The adequate concentration of GM-CSF needs to be titrated to find the optimum concentration for BMDCs differentiation as examined by flow cytometry and response to stimulation with PAMPs or DAMPs. Supernatant from GM-CSF producing cells used for BMDC culture^4^^,^^5^ can be replaced by commercially available GM-CSF. If using this method, choose a preparation with low endotoxin content.

### Supernatant generation

**Timing: 1 day**

2. Harvest BMDCs at d7 or d8 and generate stimulatory SN.
  ***Note:*** Before starting BMDC harvesting, warm medium to 37°C. Harvesting BMDCs yields approximately 6**–**12 × 10^6^ cells per petri dish but may vary between individual mice.
  ***Optional:*** BMDCs can also be seeded and stimulated in 24-, 12- or 6-well plates, with the cell number and volume of cell culture medium increasing accordingly.
  a. To harvest loose and semi-adherent cells, transfer 15 mL medium from the petri dish into a 50 mL tube (the total volume of medium in the petri dish at the time of harvesting is approximately 30 mL).
  b. Section the petri dish into four quarters and rinse each quarter five times with remaining medium, use a 10 mL serological pipette and the pipette controller at maximum power.
    ***Note:*** The pipet controller should be charged fully prior to harvesting the BMDCs.
    After rinsing, collect the remaining cell suspension and pool all harvested cells. Adherent cells are discarded with the Petri dish.
  c. Centrifuge (8 min, 300 × *g*, room temperature).
  d. Discard the supernatant.
  e. Resuspend the cells in 1 mL medium, then add 9 mL of medium.
  f. Count cells at a 1:10 dilution.
  g. Adjust volume of the cell suspension to a density of 5 × 10^5^ cells/mL.
  h. Plate 100 μL of cell suspension (equals 5 × 10^4^ cells) per well into a flat bottom 96-well plate.
    ***Note:*** During seeding, gently shake tube regularly to ensure homogenous distribution of cells in the suspension.
  i. Rest cells in the incubator for 3**–**4 h.
  j. Stimulate cells for 18 h with the DAMP or PAMP of interest, e.g., add BPI at a final concentration of 200 nM (to generate SN BPI).
    ***Note:*** The final volume per well should be 200 μL, thus, the amount of added stimulation solution should be 100 μL. Recombinant expressed BPI as well as commercially available BPI can be used.^1^
  k. Harvest SN.
    ***Note:*** Avoid absorbing any cells during the harvesting process.
  l. Centrifuge SN after the harvesting process (8 min, 300 × *g*, room temperature) to ensure no cells remain in the suspension. SN are stored at −20°C.
3. Control quality of BMDC differentiation.
  ***Note:*** Flow cytometry analysis of CD11c and CD11b expression can be used to control for the rate of adequately differentiated BMDCs. All staining should be performed on ice and with cold buffer (4°C).
  a. Transfer cells into tubes suitable for flow cytometry.
  b. Centrifuge (8 min, 300 × *g*, 4°C).
  c. Discard supernatant.
  d. Dilute Fc block (αCD16/αCD32, final dilution: 1:250) in FACS buffer.
  e. Resuspend cells in 50 μL FACS buffer containing Fc block (final concentration: 2 μg/mL) and gently vortex suspension.
  f. Incubate for 10 minutes in the dark.
  g. Prepare an antibody master mix of CD11c-Vioblue (final dilution 1:50) and CD11b-VioGreen (final dilution 1:50).
    ***Note:*** Use FACS buffer for antibody dilution.
  h. Stain cells by adding 50 μL master mix and vortex gently.
  i. Incubate for 30 minutes in the dark.
  j. Add 500 μL FACS buffer.
  k. Centrifuge (8 min, 300 × *g*, 4°C).
  l. Discard supernatant.
  m. Resuspend cells in 200 μL FACS buffer.
  n. Add 5 μL of 7AAD-solution 5 min ahead of measurement and vortex gently.
    ***Note:*** The 7AAD negative population should contain >65% CD11c^+^CD11b^+^ cells when analyzed on d7. To increase cell yield, please refer to Problem 2 in the troubleshooting section.

### CD4^+^ T cell isolation

**Timing: 3 h**

The procedure of murine CD4^+^ T-cell isolation from spleen and peripheral lymph nodes is described hereafter.

***Note:*** Before starting CD4^+^ T-cell isolation, cool sterile PBS and the centrifuge to 4°C. If cells are isolated from spleen, prepare red blood cell lysis buffer.

***Optional:*** Besides cells from spleen and/or peripheral lymph nodes, cells from mesenterial lymph nodes can also be isolated accordingly. For the isolation of cell types other than (naïve: CD25^-^CD44^-^CD62L^+^) CD4^+^ T cells from C57BL6/J or other mouse strains, please refer to Problem 3 in the troubleshooting section.

4. Sacrifice 8**–**10 weeks old male C57BL/6J mice by carbon dioxide asphyxiation or cervical dislocation.

***Note:*** Usage of older mice might influence results.

5. Sterilize the abdominal region using 70% EtOH.
6. Harvest spleen and peripheral, i.e., inguinal and axillar, lymph nodes.

***Note:*** Use sterilized instruments for harvesting organs as described for BMDC generation above.

***Optional:*** In addition to inguinal and axial lymph nodes, cervical, brachial and popliteal lymph nodes may be harvested.

7. Store organs in 500 μL PBS on ice.

***Note:*** Work in a laminar air flow cabinet for all subsequent steps.

**Figure 1:**
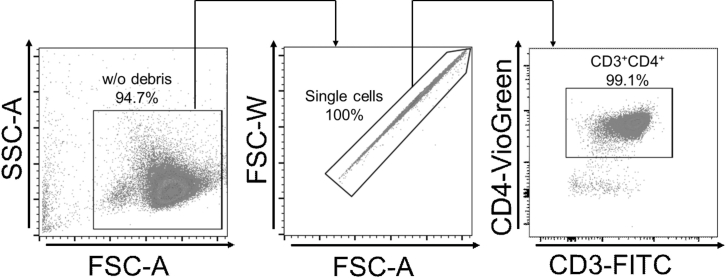
Flow cytometric quality control of CD4^+^ T-cell sort via MACS technique

8. Homogenize and strain organs.
  a. Prepare a 70 μL cell strainer, the plunger of a 5 mL syringe and a 50 mL tube.
  b. Put the cell strainer onto the tube and pre-soak it with the PBS.
  c. Put organ(s) onto the cell strainer.
  d. Mechanically pass the tissue through the cell strainer using the plunger.
  e. Rinse the plunger with 1 mL PBS into the tube.
  f. Wash cell strainer three times with 1 mL PBS each.
  g. Centrifuge (8 min, 300 × *g*, 4°C).
9. Lyse erythrocytes.
  ***Note:*** This step is required for cells harvested from the spleen and not required if harvested from lymph nodes.
  a. Following step 5g, discard supernatant.
  b. Resuspend cell pellet in 1 mL of red blood cell lysis buffer.
  c. Incubate for 1 min on ice while mixing gently every 15 sec.
    ***Note:*** Small visible cell aggregates should form in this step.
  d. Add 10 mL of PBS.
  e. Centrifuge (8 min, 300 × *g*, 4°C).
  f. Discard supernatant.
  g. Resuspend the cells in 1 mL of PBS, then add 9 ml of PBS.
  h. Centrifuge (8 min, 300 × *g*, 4°C).
    ***Note:*** Please be aware that after step 6g, larger cell aggregates may form in the suspension. For guidance on how to address this, please refer to Problem 4 in the troubleshooting section.

10. Prepare cells for negative cell sort.
  ***Note:*** This section was adopted from the manufacturer’s protocol (CD4^+^ T-cell isolation Kit, Miltenyi, Order no. 130-104-454; https://static.miltenyibiotec.com/asset/150655405641/document_nufdi4ohkh571c6d2b2atbim6n?content-disposition=inline). All steps should be performed on ice and with MACS buffer cooled to 4°C.
  a. Discard supernatant.
  b. Resuspend the cells in 1 mL of MACS buffer, then add 9 mL of MACS buffer.
  c. Count cells at a 1:10 dilution.
  d. Centrifuge (8 min, 300 × *g*, 4°C).
  e. Discard supernatant.
  f. Add 40 μL MACS buffer per 10^7^ cells (e.g., 120 μL MACS buffer for a total of 3 × 10^7^ cells).
  g. Add 10 μL of biotinylated antibody mix of the Miltenyi CD4^+^ isolation kit per 10^7^ cells and vortex gently.
  h. Incubate for 5 min on ice.
  i. Add 30 μL MACS buffer per 10^7^ cells.
  j. Add 20 μL anti-biotin microbead solution of the Miltenyi CD4^+^ isolation Kit per 10^7^ cells and vortex gently.
  k. Incubate 5 min on ice.

11. Negative MACS sort of CD4^+^ cells (Yield ≈ 10% of input).
  ***Note:*** This section was adopted from the manufacturer’s protocol (CD4^+^ T-cell isolation Kit, Miltenyi, Order no. 130-104-454; https://static.miltenyibiotec.com/asset/150655405641/document_nufdi4ohkh571c6d2b2atbim6n?content-disposition=inline).
  a. Prepare the MidiMACS separator (sterilize for usage in the lamina air flow).
  b. Insert LS column into the magnetic separator.
    ***Note:*** For lymph node cells only, a MS column might be sufficient.
  c. Equilibrate column using 3 mL MACS puffer.
    ***Note:*** Do not let the column run dry while performing the next steps.
    **CRITICAL:** During the separation you must collect and combine all flow-through in a fresh 50 mL tube.
  d. Add cell solution and let it run into the column.
  e. Flush column 5 times with 1 mL MACS buffer each.
    ***Note:*** The cells of interest are in the flow-through, other cells are bound in the column.
    **CRITICAL:** Do not remove the column from the magnetic separator while separation. Do not discharge any flow-through while the separation column is in the separator.
  f. Centrifuge (8 min, 300 × *g*, 4°C) the tube with all collected and combined flow-through.
  g. Discard supernatant.
  h. Resuspend the cells in 1 mL medium (or MACS puffer for further naïve T-cell isolation), then add 9 mL medium (or MACS buffer).
  i. Count cells (no dilution needed).
    ***Optional:*** The purity of the separated cells can be assessed via flow cytometry analysis by staining and gating for CD3^+^CD4^+^ cells (see Figure 1). Purity is typically greater than 97%.

### Naive CD4^+^ T cell isolation

**Timing: 2 h**

The procedure of naïve murine CD4^+^ T-cell isolation is described hereafter.

***Note:*** This is an optional additional step to further separate naïve cells. If isolating the CD4^+^ cells is sufficient for the experiment, skip this part and move directly to “CD4^+^ T-cell stimulation”. Work in a laminar air flow cabinet for subsequent steps.

12. Stain cell surface for fluorescence-activated cell sorting (FACS; Yield ≈ 30**–**50% of input).
  ***Note:*** If no FACS device is available, the sorting of naïve T cells can be performed using a naïve MACS Sort Kit from Miltenyi Biotec (130-104-453 ). Sorting should be performed according to the manufacturer’s protocol (https://static.miltenyibiotec.com/asset/150655405641/document_nufdi4ohkh571c6d2b2atbim6n?content-disposition=inline).
  a. Centrifuge CD4^+^ T-cell suspension (8 min, 300 × *g*, 4°C).
  b. Discard supernatant.
  c. Resuspend the cells in 100 μL MACS buffer per 2 × 10^6^ cells.
  d. Transfer cell solution into a flow cytometry tube.
  e. Stain cells for CD4-PerCP (final dilution 1:100), CD62L-PE (final dilution 1:200), CD44-FITC (final dilution 1:200), and CD25-APC (final dilution 1:50) using the antibodies as indicated in the Key resources table.
    ***Note:*** Use MACS buffer for antibody dilution.
    ***Note:*** Antibodies labeled with alternative fluorochrome conjugates may be used in step 12e. Please refer to Problem 5 in the troubleshooting section.
  f. Incubate cells for 30 min on ice and in the dark.
  g. Add 500 μL PBS.
  h. Centrifuge (8 min, 300 × *g*, 4°C).
  i. Discard supernatant.
  j. Resuspend the cells in 1 mL PBS.
  k. Use a 30 μL pre-separation filter placed on a fresh FACS tube to remove cell clumps.
13. Gating strategy.

**Figure 2:**
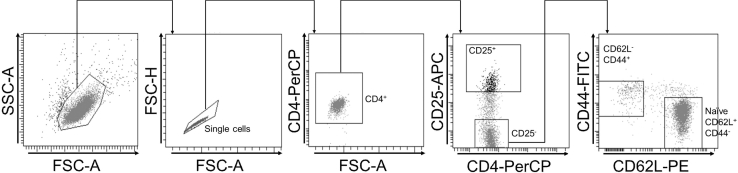
Gating strategy for further isolation of CD4^+^ T-cell populations via fluorescence-activated cell sorting

***Note:*** Cell sort for naïve T cells is done by selecting for CD4^+^CD62L^+^CD44^-^CD25^-^ cells (Figure 2). Control the purity of the cells by flow cytometry analysis. Purity is typically greater than 99%.

### T cell cultivation and stimulation

**Timing: 1–5 days**

Polyclonal T-cell activation and stimulation with BMDC-derived SN is described hereafter.

***Note:*** Before starting T-cell stimulation, warm medium to 37°C. Work in a laminar air flow cabinet for all subsequent steps.

14. Prepare flat bottom 96-well plate.
  a. Pre-coat 96-well plate with 100 μL of PBS containing αCD3 antibody at a concentration of 2.5 μg/mL for 1 h at 37°C in the incubator or overnight in the fridge.
    ***Note:*** Prior titration of αCD3 might help to improve experimental results.
  b. Discard pre-coat solution from the wells.
  c. Wash wells with 200 μL PBS.

***Note:*** Do not allow the wells run dry. Keep PBS in the wells until the cell suspension has been prepared properly.

15. Polyclonal T-cell activation and stimulation.
  a. Centrifuge (8 min, 300 × *g*, 4°C).
  b. Resuspend the cells in 1 mL medium.
  c. Adjust medium volume to achieve a cell concentration of 1.5 × 10^6^ cells/mL.
  d. Add αCD28 antibody to the cell suspension at a concentration of 8 μg/mL.
    ***Note:*** Titration of αCD28 in proceeding experiments may improve the results.
  e. Aspirate PBS from 96-well plate.
  f. Seed 100 μL cell suspension per well (equals 1.5 × 10^5^ cells per well).
  g. Add 100 μL stimulatory SN from BMDCs.
    ***Optional:*** If of interest, also add one well supplemented with medium instead of SN as a negative control. As an additional negative control, add cells without polyclonal activation. For further experiments testing stimuli other than BPI, SN BPI is used as the positive control.

### Readout

**Timing: 1–2 days**

16. Data collection and readout.
  ***Note:*** The time point at which the readout is taken can be adjusted according to the experiment; a range of days from d1 to d5 was tested. For naïve CD4^+^ T cells, a stimulation time of up to d5 is recommended.
  a. Pictures of the cells in the well can be taken daily using a reflected-light microscope in order to monitor formation of cell clusters.
  b. Cell splitting is recommended at d3 (1:2).
    i. Loosen T cells from the plate by pipetting the cell suspension five times up and down.
      ***Note:*** The total volume at d3 will be around 180 μL.
    ii. Transfer each 90 μL cell suspension into fresh wells (not pre-coated).
    iii. Add 90 μL of the BMDC-derived stimulatory SN used for stimulation.
      ***Note:*** Repetition of polyclonal activation with αCD3 and αCD28 is not required.
      ***Optional:*** In case you use an additional medium control, add 90 μL of medium.
  c. Harvest supernatant between d1 and d5 depending on your experimental objective.
    ***Note:*** Try to avoid absorbing cells.
    ***Optional:*** Analysis of secreted T-cell cytokines by ELISA or multiplex analysis are recommended. More details about the multiplex method can be found in Bülow et al.^1^⁠
  d. Harvest cells.
    ***Optional:*** Count cells and conduct intra- and extracellular flow cytometry analysis. For example, stain cells extracellular using CD3-FITC (final dilution 1:50), CD4-VioGreen (1:50), CD62L-VioBlue (final dilution 1:10), CD44-PE-Cy7 (final dilution 1:10), and CD25-APC (final dilution 1:50) as indicated in the Key resources table.

## Expected outcomes

**Figure 3:**
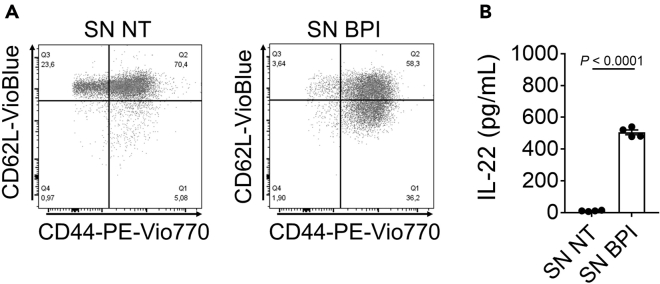
Expected results of this protocol Naïve CD4^+^ T cells cultured for d5 in supernatant of untreated BMDCs (SN NT) or in supernatant of BPI-treated BMDCs (SN BPI). (A) Representative dot blot of flow cytometric analysis of CD62L and CD44 cell surface presentation. (B) IL-22 secretion measured by Luminex technology, n = 4. Data are shown as means ± SEM. Statistical testing was performed using Student`s ratio paired *t* test.

This protocol describes the isolation of (naïve) CD4^+^ T cells from the lymph nodes and spleen of C57BL6/J mice. Supernatants (SN) from BMDCs, which have been harvested in advance, are used in combination with polyclonal activation (αCD3 and αCD28) to activate and differentiate (naïve) CD4^+^ T cells. This setup enables the investigation of the BMDC T-cell axis in the context of the initiation of adaptive immune responses following BMDC activation via DAMPS or PAMPs. As demonstrated by Bülow et al.^1^, d7 or d8 BMDCs are either left untreated (NT) or stimulated with bactericidal/permeability increasing protein (BPI, 200 nM) for 18 h, after which the harvested supernatants are stored at −20°C until use. Naïve CD4^+^ T cells are generated according to this protocol and cultured upon polyclonal activation in combination with SN derived from BMDCs. Data analysis is performed after d5 of cell culture. As an example, flow cytometry analysis as well as IL-22 cytokine secretion are shown in Figure 3. Compared to SN NT-cultured cells, SN BPI culture enhances the surface presentation of CD44 and significantly induces IL-22 secretion. IL-22 is an important cytokine for maintaining intestinal tissue homeostasis and inducing epithelial regeneration following intestinal tissue injury.^6^^,^^7^

## Limitations

This protocol enables the investigation of the BMDC T-cell axis, focusing on the activation of CD4^+^ T cells when adaptive immune responses are initiated following the activation of BMDCs via DAMPs or PAMPs. Although not shown here, the CD4^+^ cells can be replaced with CD8^+^ T cells. BMDCs were stimulated with BPI but other stimuli, such as other DAMPs or PAMPs like toll-like and C-type lectin receptor ligands, can also be employed. The interaction of the T-cell receptor with MHCI or MHCII is simulated via αCD3, and co-stimulatory signals are provided by αCD28 antibodies. Thus, there is no direct interaction between BMDCs and T cells in the cell culture setup, and the results focus on the effects of soluble factors in the cell culture supernatant. However, as described by Bülow et al.^1^, direct co-culture of BMDCs and CD4^+^ T cells is possible when the T cells are derived from mice with an antigen-specific transgenic TCR and the corresponding antigen is present. For instance, BMDCs derived from C57BL/6J mice can be pre-incubated with ovalbumin peptide 323**–**339 prior to the addition of ovalbumin-specific CD4^+^ T cells derived from OTII mice (official name: B6.Cg-Tg(TcraTcrb)425Cbn/J).

BMDC cultures supplemented with GM-CSF do not result in a mono-culture but are rather a mixture of DCs and MΦ.^2^ Depending on the scientific question, supernatants of antigen-presenting cells other than BMDCs can also be tested.

## Troubleshooting

### Problem 1

Increased background activation (related to “BMDC isolation and differentiation”).

BMDCs are highly sensitive to microbial ligands,^8^^,^^9^ which can unintentionally lead to increased background activation. This effect becomes particularly apparent when measuring elevated cytokine secretion in untreated BMDC supernatants (SN NT) and/or increased levels of maturation markers such as CD40, CD80 and CD86 on the surface of the untreated BMDCs. To avoid background activation, all of the following solutions should be implemented consistently throughout the entire protocol.

### Potential solution

Reduce the risk of contamination by bacteria or bacterial products such as lipopolysaccharide (LPS) during the harvesting of organs from mice and the preparation of BMDCs.

• Sterilize dissection instruments using dry heat at 180°C.
• Disinfect hands and work surfaces before starting.
• Wear gloves and lab coats with cuffs.
• Thoroughly disinfect the fur of euthanized mice before collecting the organs.
• Perform all steps under a laminar air flow device.
• Choose media and FCS that have been tested for low endotoxin content.

### Problem 2

Inadequate differentiation of BMDCs (related to step 3).

BMDC differentiation is considered inadequate if fewer than 65% of the cells are CD11c^+^CD11b^+^ on d7, or if the total number of cells on d7 is ≤ 4 × 10^6^ cells per plate.

### Potential solution

To improve the quality of BMDC differentiation, the following optional optimization steps can be considered.

• Extended incubation time (e.g., incubating for d8 instead of d7).
• Ensure appropriate GM-CSF titration.
• Screen multiple FCS lots to identify optimal conditions for BMDC differentiation.
• Ensure that the bone marrow is not exposed to ethanol or other harmful chemicals.
• Avoid delay during harvesting and seeding of bone marrow cells.

### Problem 3

Sort of other cells than (naïve) CD4^+^ T cells or the use of other mouse strains (related to “CD4^+^ T-cell isolation”).

This procedure is described for (naive) CD4^+^ T cells derived from C57BL/6J mice. However, the protocol can also be applied to CD8^+^ T cells and other T-cell subtypes. This procedure is additionally suitable for T cells derived from other mouse strains.

### Potential solution

• To analyze (naïve) CD8^+^ T cells, use the murine “naïve CD8a^+^ T-Cell Isolation Kit” from Miltenyi Biotech (130-096-543) in steps 10 and 11. This kit enables the isolation of total CD8^+^ T cells by negative selection and, with the addition of an included antibody also naïve CD8^+^ T cells. If the resulting purity of the naïve cells is insufficient, the additional purification described in steps 12 and 13 can be applied.
• Other mouse strains can be used if a different genetic background to that of C57BL/6J mice is desired. For example, strains such as BALB/c mice can be used.

### Problem 4

Visible cell aggregates in the lymphocyte preparation (related to step 6).

Erythrocyte lysis is performed prior to isolating T cells from the spleen. After using erythrocyte lysis buffer, the cells are washed with PBS and centrifuged. After discarding the supernatant, 1 mL of PBS is added and visible cell aggregates will appear during cell resuspension in step 9g. Follow the steps below to remove the aggregate(s).

### Potential solution

To remove large aggregates, use a 1 mL tip and the corresponding pipette to pipette 1 mL of PBS up and down five to six times. The cells will then resuspend and one (or more) large aggregate(s) of cell residues will form. We recommend using the pipette to draw out and discard the remaining aggregate(s) from the suspension.

### Problem 5

Use of antibodies labeled with alternative fluorochrome conjugates (related to step 12).

Flow cytometry analysis can be performed using different fluorochrome conjugates if required. These are chosen based on flow cytometer compatibility or reagent availability.

### Potential solution

The fluorochrome choices may differ between antibody panels. Therefore, adapt the gating strategy in step 13 accordingly.

## Resource availability

### Lead contact

Further information and requests for resources and reagents should be directed to and will be fulfilled by the lead contact, Sigrid Bülow (sigrid.buelow@ukr.de).

### Technical contact

Technical questions on executing this protocol should be directed to and will be answered by the technical contact, Christina Pfab (christina.pfab@ukr.de).

### Materials availability

Materials generated in this study will be made available on request from the lead contact but may require a completed material transfer agreement.

### Data and code availability

This paper does not report original code. All data generated during this study are included in the manuscript. Additional information can be found in Bülow et al.^1^

## Acknowledgments

We thank Lisa Reinstein, Martina Toelge, Maren Werner, Nicole Ritter, and Alexandra Müller (all from the Institute of Medical/Clinical Microbiology and Hygiene, University Hospital Regensburg, Germany) as well as Irina Fink (Leibniz Institute for Immunotherapy, University Hospital Regensburg, Germany) for their technical assistance. Furthermore, we thank Dr. Joachim Gläsner (Institute of Clinical Microbiology and Hygiene, University Hospital Regensburg, Germany) for advice and discussions.

In addition, we thank Prof. Dr. Petra Hofmann (Department of Internal Medicine III, University Hospital Regensburg, Germany) for sorting naive T cells. This work was supported by the Bavarian Ministry of Economic Affairs, Regional Development and Energy (project no. M4-2506-0005 to S.B.) and the Deutsche Forschungsgemeinschaft (DFG, project number 324392634-TRR 221(B13) to A.G.). The graphical abstract was created using Biorender.com.

## Author contributions

Conceptualization, S.B.; methodology, K.U.R., S.B., J.H., and C.P.; visualization, C.P.; writing – original draft, C.P.; writing – review and editing, all authors; funding acquisition, A.G.; supervision, S.B.

## Declaration of interests

J.H. is now a full-time employee of FUTRUE GmbH. K.U.R. is now a full-time employee of Haupt Pharma Amareg GmbH, Member of the Aenova Group. The University of Regensburg is applying for a patent (PCT/EP2019/061989) covering parts published in this manuscript with A.G. and S.B. as inventors.
